# Evaluation of Postoperative Effects of Using Ozonated Water as an Irrigant on Soft and Hard Tissues Surrounding Dental Implants: A Randomized Controlled Clinical Trial

**DOI:** 10.7759/cureus.68555

**Published:** 2024-09-03

**Authors:** Mayuri Surana, Shubha Joshi, Pronob Sanyal, Shivsagar Tewary

**Affiliations:** 1 Department of Prosthodontics, School of Dental Sciences, Krishna Vishwa Vidyapeeth, Karad, IND

**Keywords:** soft tissue healing around implants, osseointegration of dental implants, post-op effects of implant placement, dental implants, ozonated water

## Abstract

Aim

To evaluate the short-term and long-term postoperative effects of ozonated water when used as an irrigant in terms of postoperative pain, healing, and implant stability when compared to normal saline irrigation during implant surgery, both carried out using conventional drilling protocol.

Methods

A total of 34 implants were placed in 17 patients, two implants in each patient, one implant using normal saline as an irrigant and another one using ozonated water as an irrigant during the surgical procedure of implant site osteotomy. Postoperative pain was assessed after 48 hours of the surgical procedure using the visual analog scale (VAS). Soft tissue healing was assessed after eight days using the tissue healing index. Osseointegration was checked by measuring the primary stability at the moment of implant placement and comparing it to the secondary stability measured three months after the implant placement. These stability values (ISQ) were obtained using resonance frequency analysis (RFA).

Results

The VAS scores for the control group (Group A) after 48 hours were 71.76±5.57 and for the experimental group (Group B) after 48 hours were 47.64±5.33 so mean values in the experimental group were significantly lower as compared to that in the control group (p<0.001). The mean healing index score for the control group (Group A) was 3.35±0.49 and the mean healing index score for the experimental group (Group B) was 4.64±0.49 so the mean values of tissue healing index in the experimental group were significantly higher as compared to that in the control group (p<0.001). The increase in stability value over the period of three months is 5.83 ISQ in the control group while the increase in stability value over the period of three months is 7.06 ISQ in the experimental group. The difference although not statistically significant shows a slight increase in stability in the experimental group as compared to that of the control group.

Conclusion

Ozone water irrigation at implant site osteotomy reduced postoperative pain and accelerated the tissue wound healing but the significant effect on osseointegration could not be determined.

## Introduction

The inception of modern implant treatment dates back to the 1960s in Sweden, driven by the pioneering research where Branemark introduced the revolutionary concept of osseointegration, demonstrating that metallic biomaterials, particularly titanium, could integrate with bone when left in place for a specific duration. Branemark's investigations revealed a strong bond between the bone and the titanium surface, highlighting biocompatibility as a crucial biological property of the metal [1].

Oral implantology, while typically not life-threatening, is a frequently performed procedure associated with both physical and psychological impacts. Despite its commonality, the experience can still induce stress and act as a deterrent on the lookout for dental care. The pain subsequent to the placement of dental implants is often characterized as mild to moderate. It is widely acknowledged that the presence of anxiety heightens sensitivity to pain, particularly during events like dental implant placement [2].

Pain following dental implant placement is a prevalent occurrence primarily resulting from intraoperative trauma and the release of pain mediators through the surgical procedure. The peak of pain typically occurs within three to five hours after surgery, overlapping with the diminishing effects of the local anesthetic used during the procedure [3].

Typically, swelling after dental implant placement peaks within the initial 72 hours and progressively diminishes over the subsequent days, completely subsiding between eight to ten days post-surgery. Management of swelling often involves the use of oral analgesics or non-steroidal anti-inflammatory drugs (NSAIDs). However, certain analgesics exhibit noticeable side effects including gastrointestinal irritation, systemic bleeding tendency, and allergic reactions [4].

The success of functional dental implants depends on successful osseointegration. Osseointegration unfolds in two distinct stages: primary and secondary. It is generally measured in terms of implant stability. Primary stability relies on the initial mechanical integration of the implant with the bone following its placement. In contrast, secondary stability is achieved through bone regeneration and remodeling. The latter synonymous with biological stability is crucial for ensuring the long-term success of the dental implant [5].

To enhance the aforementioned characteristics pertaining to dental implant placement, several conjunctive treatment modalities have been utilized. These include the application of cryotherapy, low-level laser therapy, ultrasound, osseodensification, the application of blood analogs, and other such methods aimed at improving the overall outcome of the surgical procedure. Ozone therapy is one such emerging treatment modality [6].

Ozone therapy is defined as a versatile bio-oxidative therapy, in which ozone is administered via gas or dissolved in water or oil base to obtain therapeutic benefits [7].

Ozone therapy has evolved to become a significant component in the treatment of infection, pain, swelling, inflammation, and musculoskeletal spasms. It is now applied in various fields such as surgery, oncology, dermatology, cosmetics, and dentistry [8].

After reviewing the literature, we found that more substantial and rigorous evidence is necessary before ozone can be considered suitable for use in surgical procedures. This study, therefore, evaluates the postoperative effects of using ozonated water during implant surgery. The aim of the present study is to assess the short-term and long-term postoperative effects of ozonated water, used as an irrigant, on postoperative pain, healing, and implant stability, compared to normal saline irrigation during dental implant surgery, both conducted using a conventional drilling protocol.

## Materials and methods

Subjects

**Figure 1:**
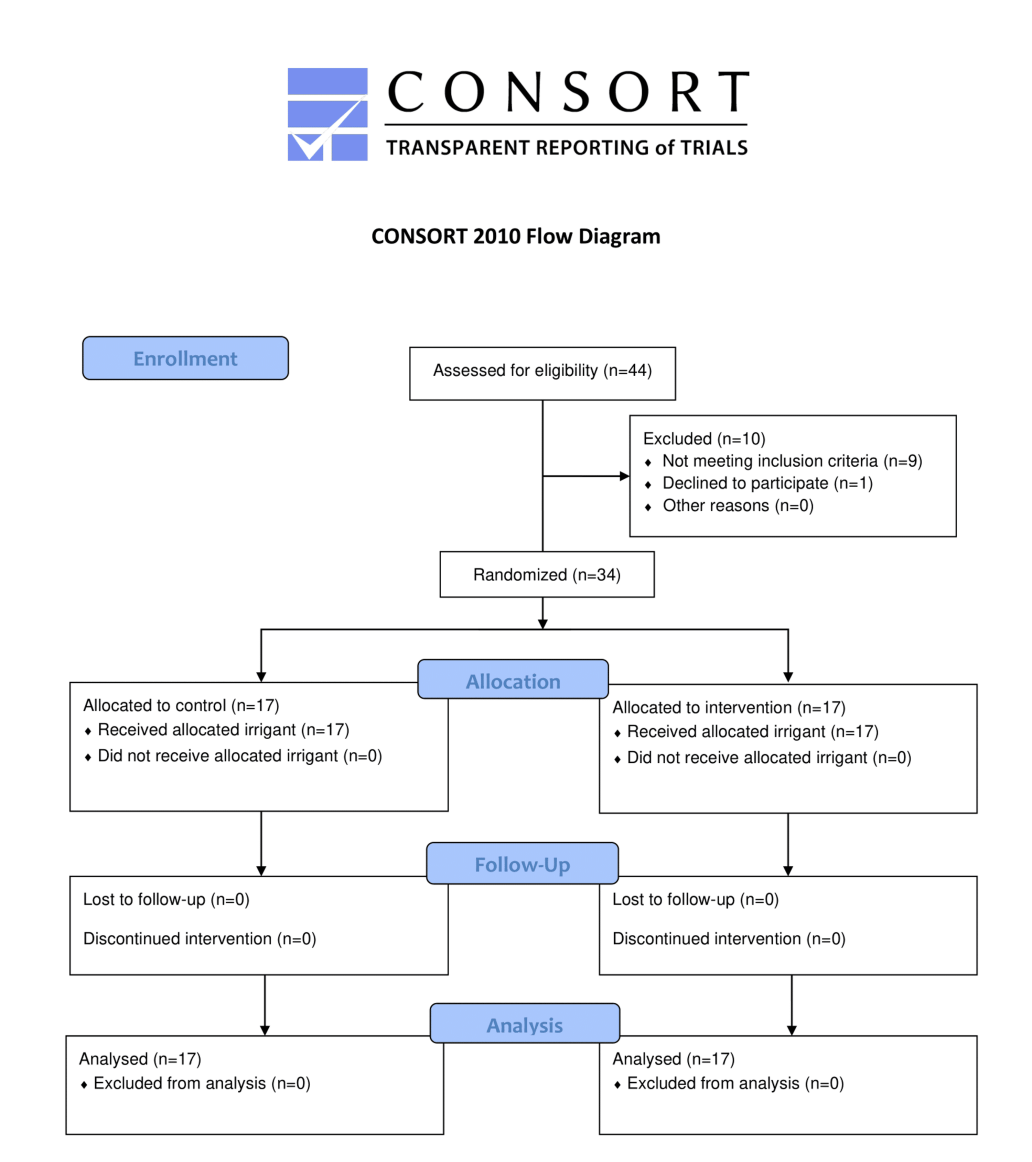
CONSORT flow diagram Moher D, Hopewell S, Schulz KF, et al. CONSORT 2010 explanation and elaboration: updated guidelines for reporting parallel group randomised trials. Br Med J. 2010 Mar 24;340. Permission to reproduce is obtained under the terms of the Creative Commons Attribution License, permitting unrestricted use, distribution, and reproduction so long as the original work is properly cited.

The present study was conducted in the Department of Prosthodontics and Crown and Bridge, School of Dental Sciences, Krishna Vishwa Vidyapeeth (KVV), Karad after due approval from the institutional protocol committee (005/2022-2033) and registration in Clinical Trials Registry of India (CTRI2024/05/067551). A minimum total sample size of 34 (17 in each group) was found to be sufficient for an alpha of 0.05, power of 80%, and drop-out rate of 10%, and a written consent was obtained from subjects, before inclusion in the study. The CONSORT diagram provides a detailed visual summary of the participant flow through the study, including the stages of enrollment, allocation, follow-up, and analysis (Figure 1).

Inclusion and exclusion criteria

Non-smoker healthy individuals or patients with controlled medical conditions requiring a minimum of one dental implant on either side of their upper or lower jaws, mean age group 30-60 years, willing to sign the consent form were included in the study. Systemically ill patients, lactating mothers or pregnant women, those requiring bone grafting or augmentation procedures, having a history of receiving radiotherapy in the head and neck region were excluded from the study. Contralateral sites were selected for implant placement in two quadrants of the same jaw. The available bone was evaluated using CBCT assessment before planning the implant surgery and sites without any bony defects were included in the study. 

Study design

This was a randomized controlled clinical trial where two implants were placed in the same patient within two weeks with one site acting as the control group and the other site as the intervention group. Randomization was conducted using a computer-generated random sequence. To prevent selection bias, allocation concealment was achieved using sealed, opaque, and alternately numbered envelopes, one site being allotted as intervention and the other as control. These envelopes were prepared by a statistician not involved in the trial and opened only after the participant's consent was obtained. Blinding was implemented as follows: participants were blinded to the group allocation. The surgeon performing the procedures was not blinded due to the nature of the intervention (different irrigants). However, the outcome assessor was blinded to the group allocation to minimize assessment bias. An interim analysis was planned after 50% of the participants completed the three-month follow-up to identify any significant differences between groups that might warrant early termination of the study for ethical reasons or to adjust the sample size if necessary. To ensure the reliability of the outcome assessments, the observer underwent training and standardization sessions before the trial commenced.

Surgical procedure

The implants were distributed into two separate groups, Group A/control group, i.e., where the osteotomy was performed using normal saline as an irrigant, and Group B/intervention group, i.e., where the osteotomy was performed using ozonated water as an irrigant.

**Figure 2:**
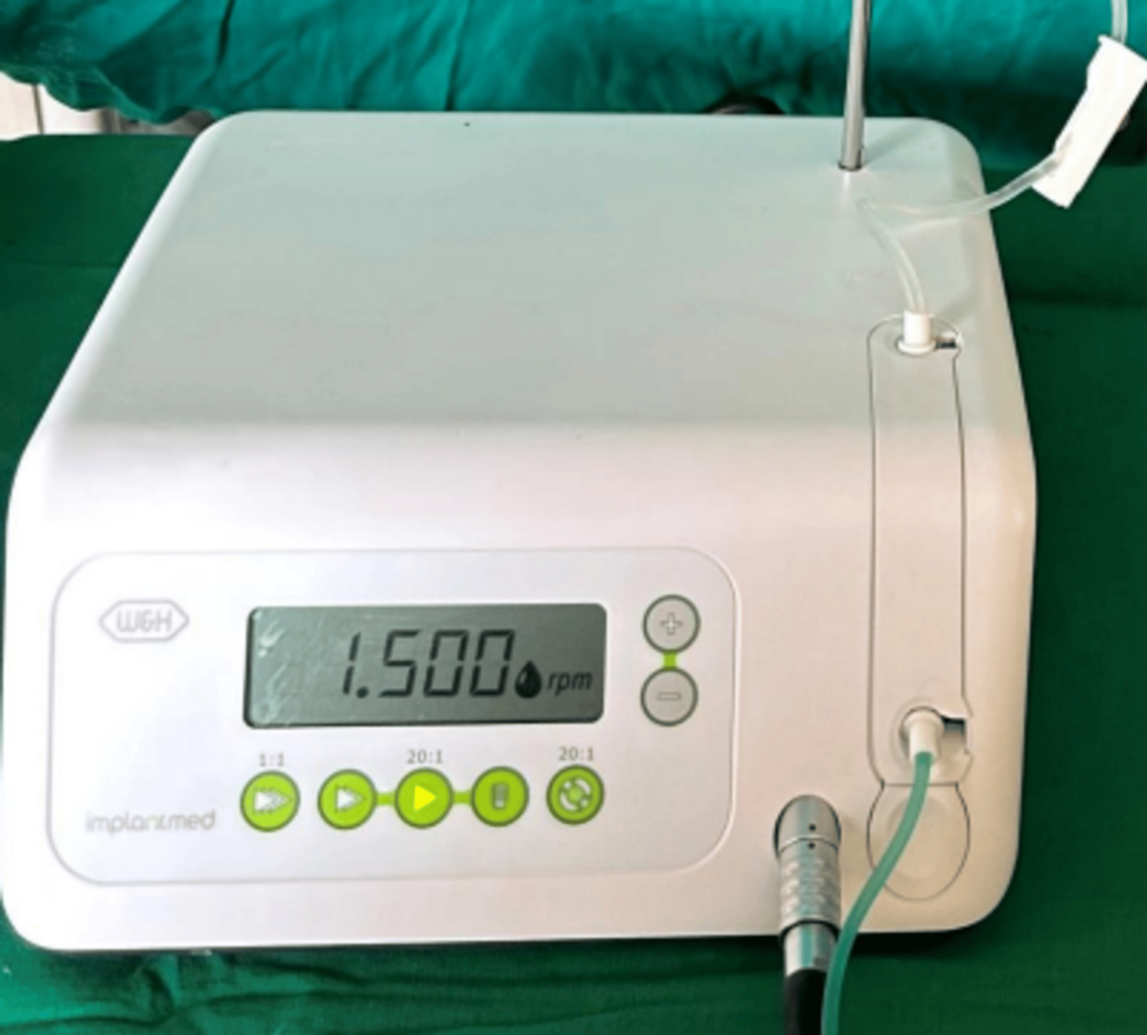
Physiodispenser

**Figure 3:**
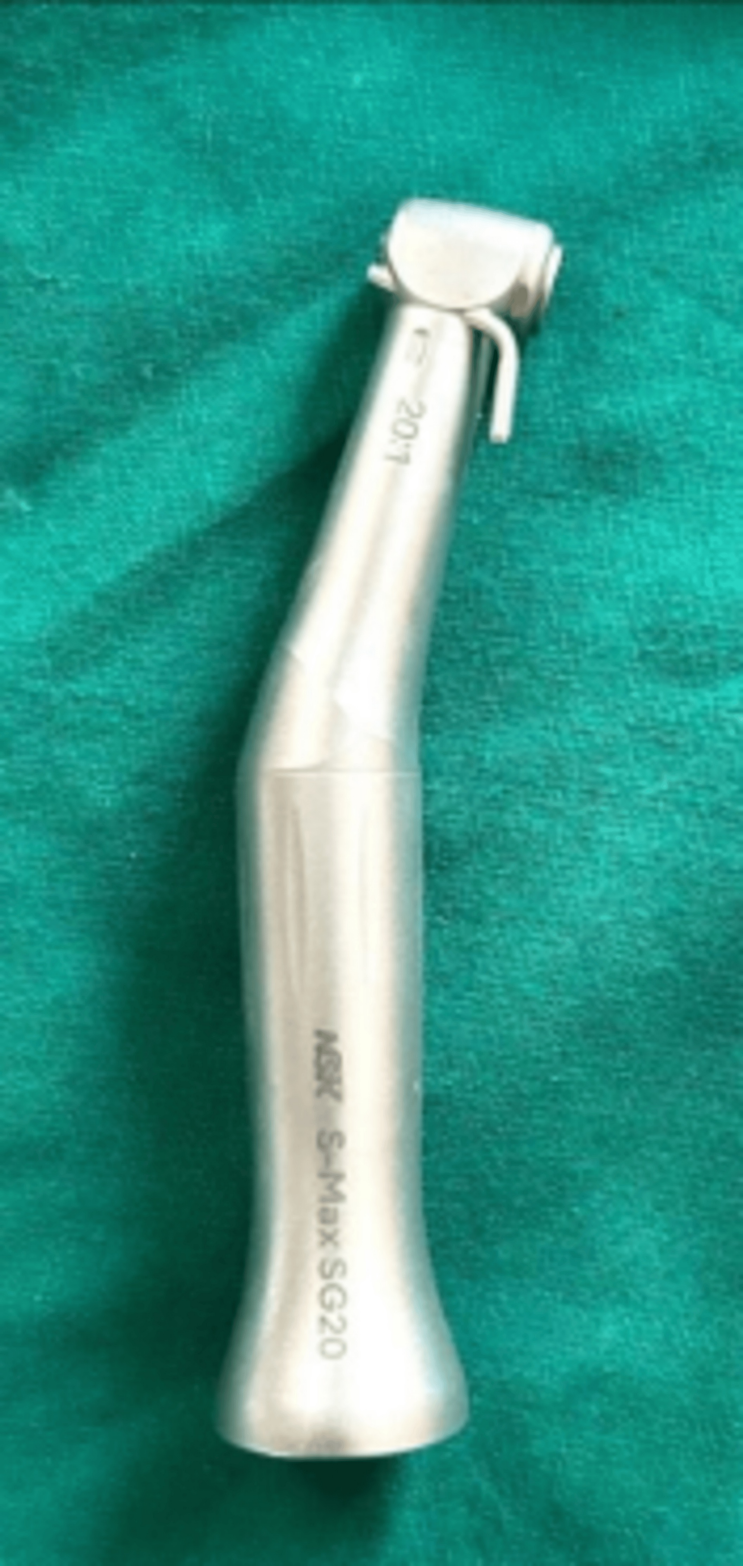
Implant surgical handpiece

All surgeries were performed under local anesthesia (2% lignocaine with adrenaline 1:80000). A surgical guide was placed and the initial lance drill was used to create a mark at the implant site. A mucoperiosteal flap was reflected and the process of implant osteotomy was initiated with the pilot drill to accurately reproduce the angulation. A series of sequential drills were used to prepare the osteotomy site precisely and incrementally to receive an implant of the appropriate size. Irrigation was constantly provided at the site of implant osteotomy to prevent overheating, which could lead to necrosis of the bone. The sites where normal saline was used were considered as the control group. The normal saline container was connected to the physiodispenser (W&H, Austria) (Figure 2) and to the NSK implant handpiece (NSK, Japan) (Figure 3) through which the saline was flowing directly into the osteotomy site. At the intervention sites, irrigation was done using ozonated water.

**Figure 4:**
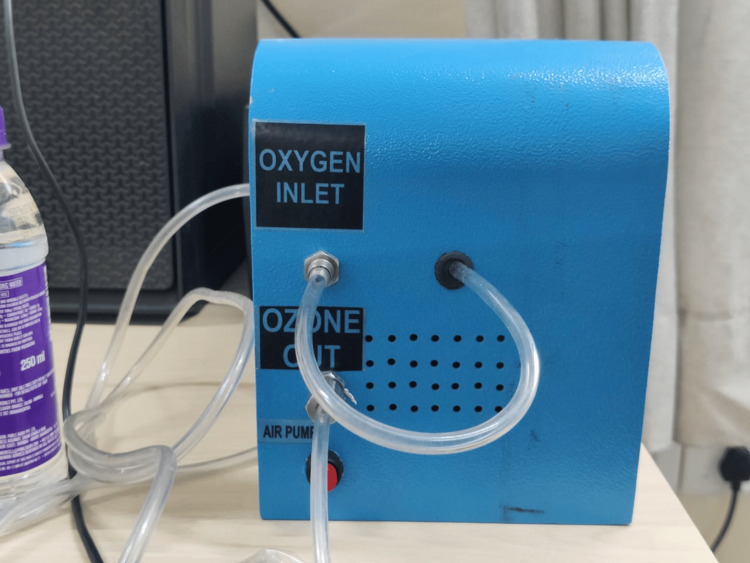
Medical-grade ozone generator

The preparation of ozonated water was carried out by using the Medical Ozone Generator (Waterhouse, India) (Figure 4). The ozone generator machine was set to emit a 35 μg/mL concentration of ozone gas.

**Figure 5:**
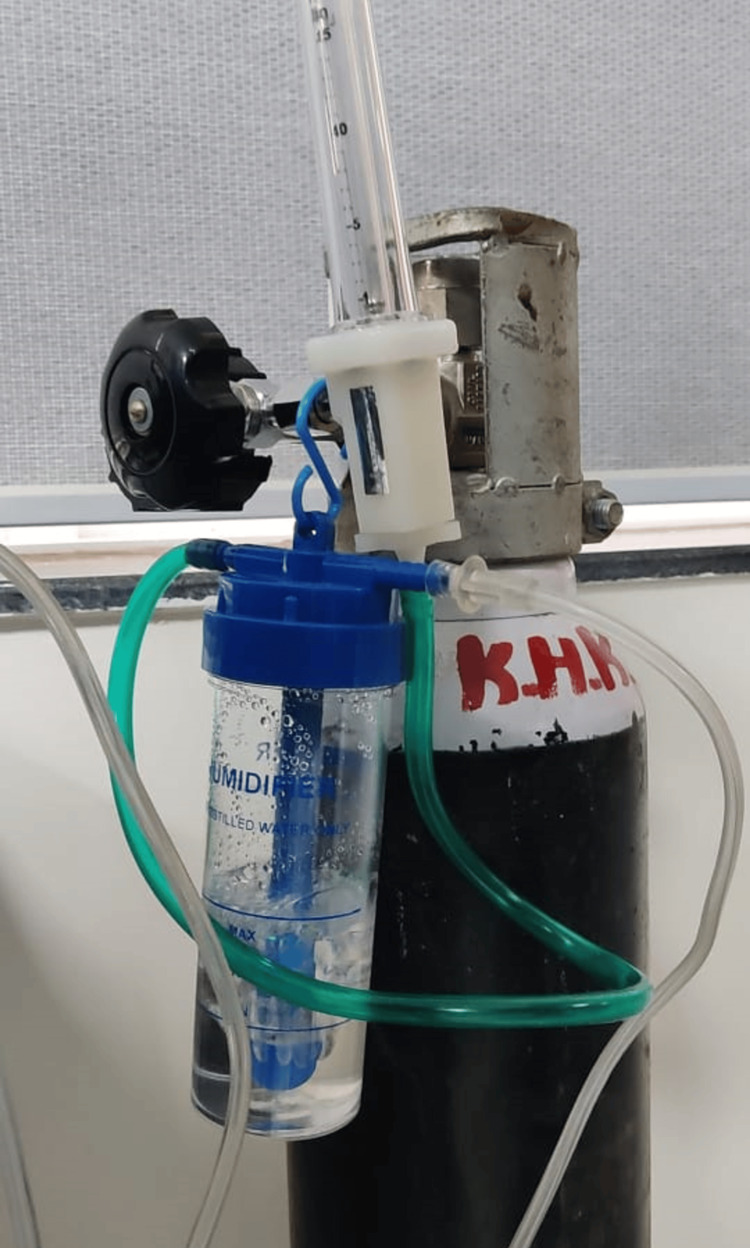
Medical-grade oxygen cylinder

**Figure 6:**
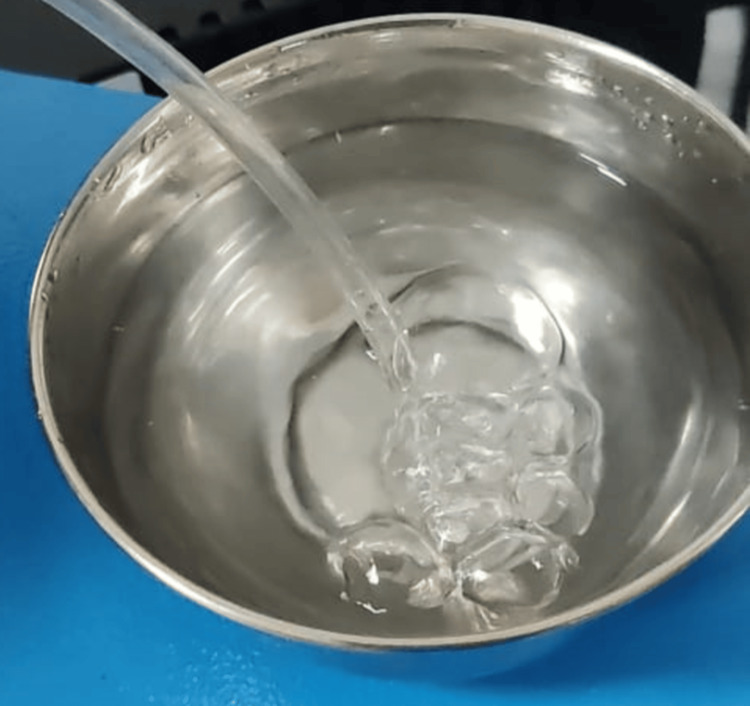
Bubbling of distilled water with ozone gas

A medical oxygen cylinder (Figure 5) was attached to the inlet nozzle of the machine through a rubber pipe. Another pipe was connected to the outlet nozzle from where the converted oxygen in the form of ozone gas was emitted by the machine. This outlet pipe was immersed in an autoclaved bowl of distilled water for seven minutes as per the manufacturer’s instructions (Figure 6).

**Figure 7:**
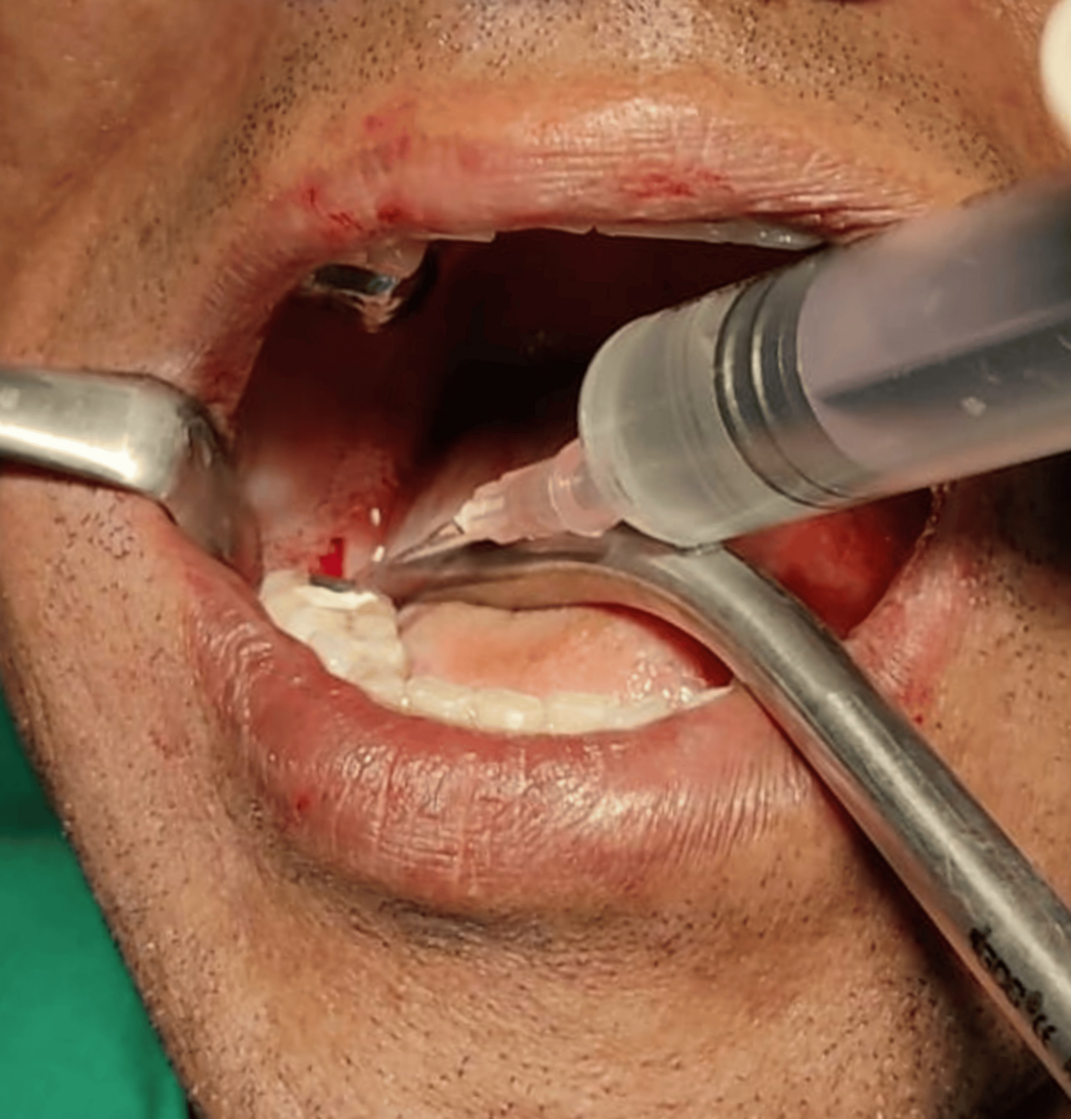
Implant site osteotomy in 46 regions irrigated by ozone water

This ozonated distilled water was used immediately by filling it in a sterile single-use syringe and manually administering it at the site of osteotomy during the procedure of drilling. The irrigation from the physiodispenser was switched off while using ozonated water as the irrigant (Figure 7). The sequential drilling protocol was the same as that used during the control group. 

**Figure 8:**
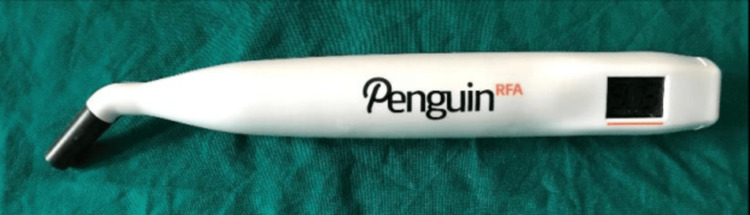
PenguinTM RFA unit RFA: resonance frequency analysis

**Figure 9:**
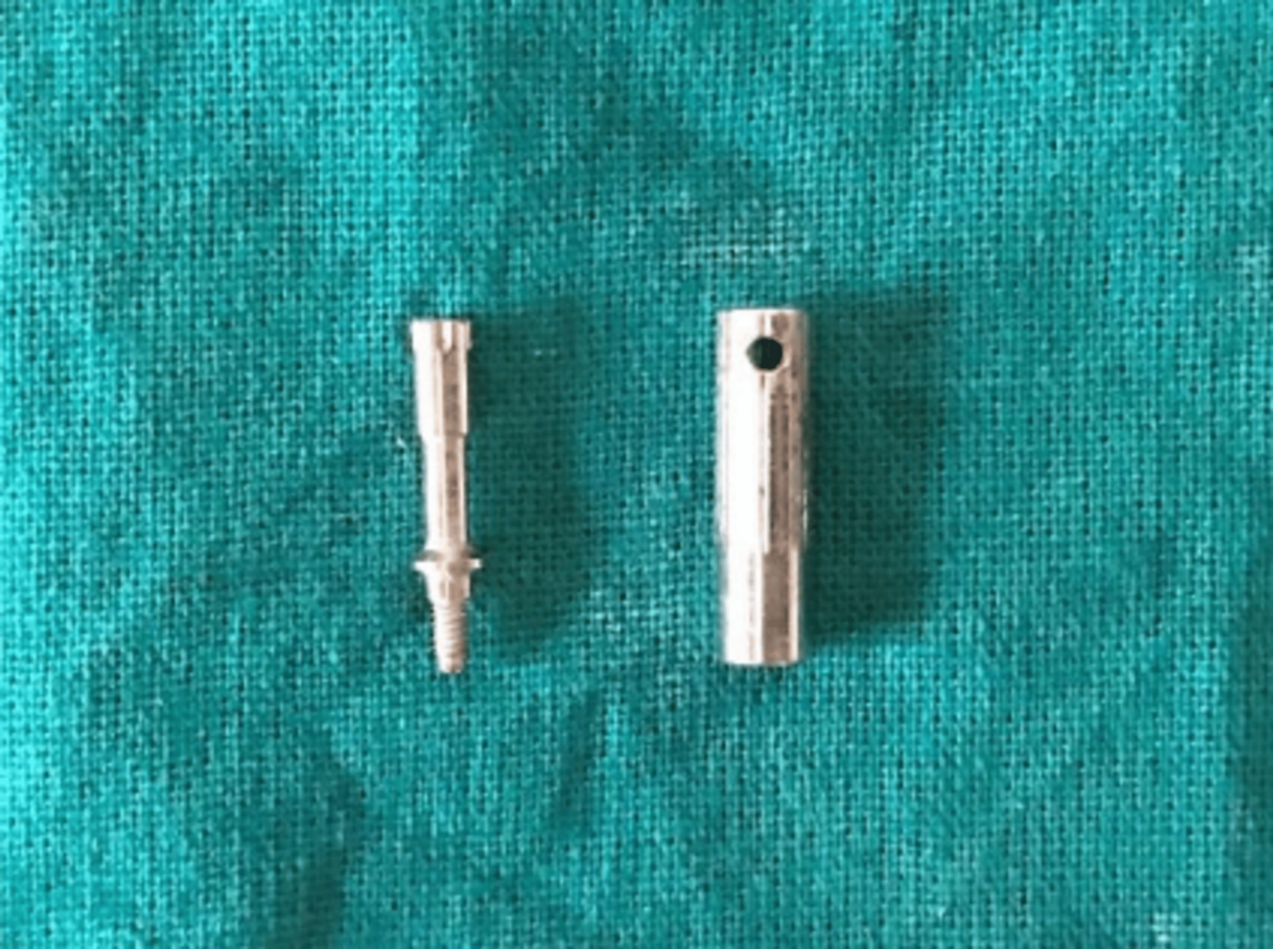
Smartpeg of RFA unit RFA: resonance frequency analysis

Once the implant placement was done, resonance frequency analysis (RFA) was performed using the Penguin^TM^ instrument (Aseptico Inc., USA) (Figure 8) by inserting a standardized abutment (Smartpeg) (Figure 9) into each implant. All readings were taken from the buccal aspect for the purpose of standardization. The values for primary stability were thus determined and noted.

The cover screws were then secured and the surgical sites were thoroughly irrigated using betadiene diluted with saline. Proper closure of the flap using 3-0 Mersilk^TM^ sutures was done aiming at maximum approximation.

Clinical analysis and outcome

**Figure 10:**
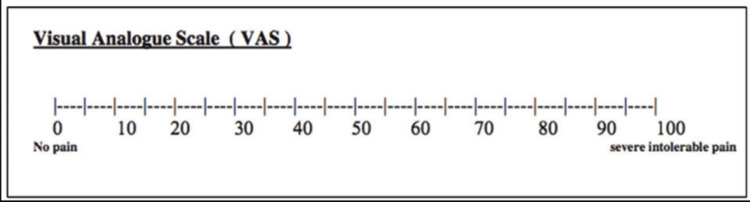
VAS VAS: visual analog scale

The patients were called for follow-up after 48 hours to assess the wound and record scores for pain using the visual analog scale (VAS) for both sites (Figure 10).

Next, the patients were called after eight days for suture removal, and assessment of wound healing and the tissue response was recorded using a standardized tissue healing index (Table 1).

**Table 1:** Tissue healing index Amaliya A, Ramadhanti R, Hadikrishna I, et al. The effectiveness of 0.2% chlorhexidine gel on early wound healing after tooth extraction: a randomized controlled trial. Eur J Dent. 2022 Jul;16(3):688-694. Permission to reproduce is obtained under the terms of the Creative Commons Attribution License, permitting unrestricted use, distribution, and reproduction so long as the original work is properly cited.

Healing Index	Criteria
Very poor (1)	Tissue color: more than 50% of gingivae red; response to palpation: bleeding; granulation tissue: present; incision margin: not epithelialized with loss of epithelium beyond margins; suppuration: present
Poor (2)	Tissue color: more than 50% of gingivae red; response to palpation: bleeding; granulation tissue: present. ; incision margin: not epithelialized with connective tissue exposed
Good (3)	Tissue color: less than 50% of gingivae red; response to palpation: no bleeding; granulation tissue: none; incision margin: no connective tissue exposed
Very good (4)	Tissue color: less than 25% of gingivae red; response to palpation: no bleeding; granulation tissue: none; incision margin: no connective tissue exposed
Excellent (5)	Tissue color: all gingivae pink; response to palpation: no bleeding; granulation tissue: none; incision margin: no connective tissue exposed

**Figure 11:**
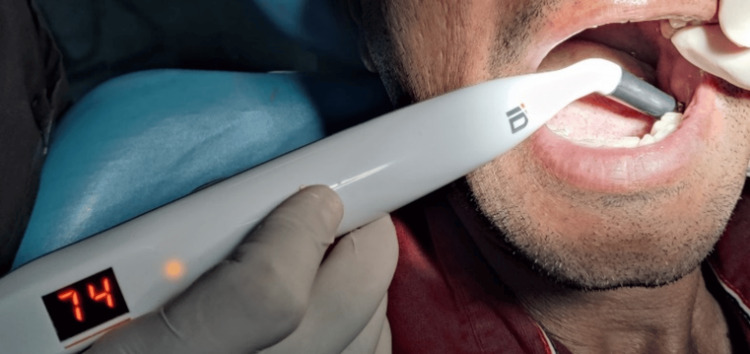
RFA measurement of control group implant site RFA: resonance frequency analysis

**Figure 12:**
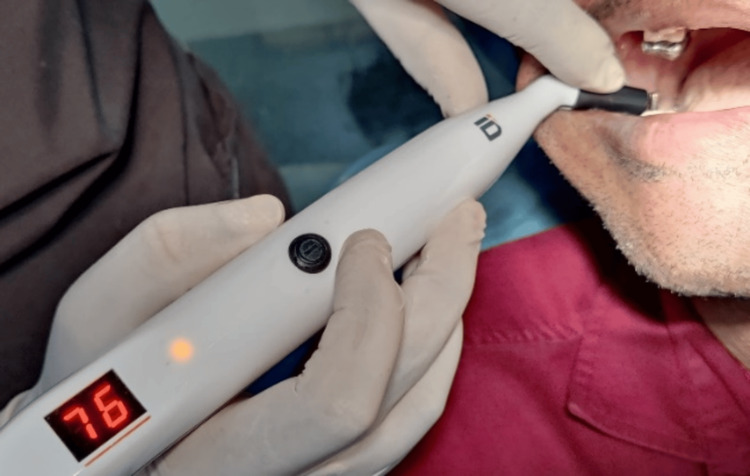
RFA measurement of the experimental group implant site RFA: resonance frequency analysis

**Figure 13:**
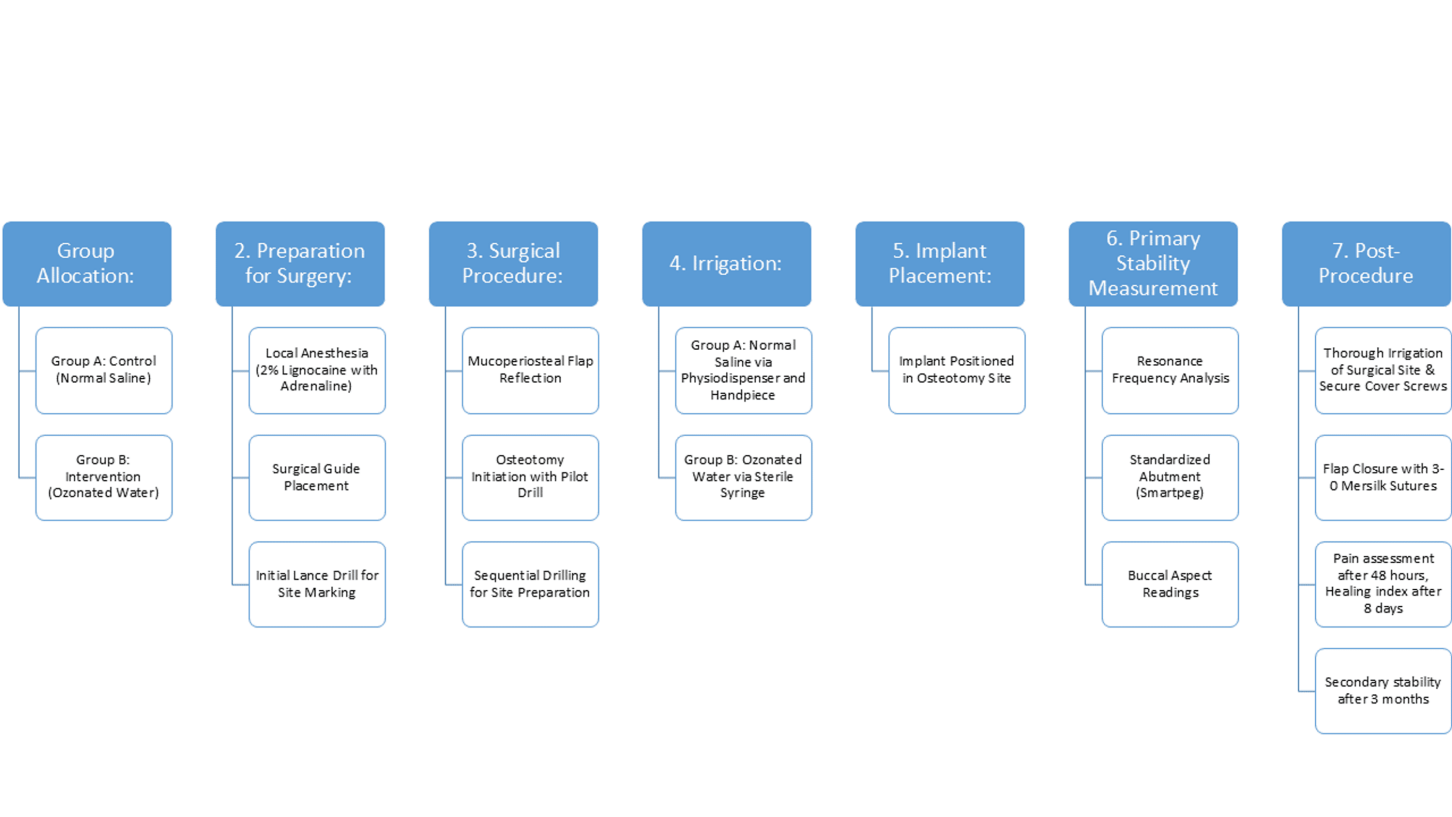
Flowchart of the study methodology

A second-stage implant exposure surgery had to be performed after the prescribed healing period of approximately three months. At this point, the secondary stability of the implant was evaluated. All readings were taken from the buccal aspect for the purpose of standardization. These ISQ values were recorded and noted as the secondary stability of the implants (Figures 11, 12). The flowchart depicting the entire study methodology has been added (Figure 13).

Statistical analysis

The samples were analyzed using the “SPSS 25.0” (IBM Corp., Armonk, NY) package, IBM, India, suggested for data analysis in the area of health sciences. The Kolmogorov-Smirnov and Shapiro-Wilk tests were performed to check if the data was normally distributed. Since the data was found to be non-normally distributed, the Mann-Whitney U test, a non-parametric test, was applied to compare the two groups, Group A and Group B.

## Results

Effect of ozonated water on postoperative pain

**Figure 14:**
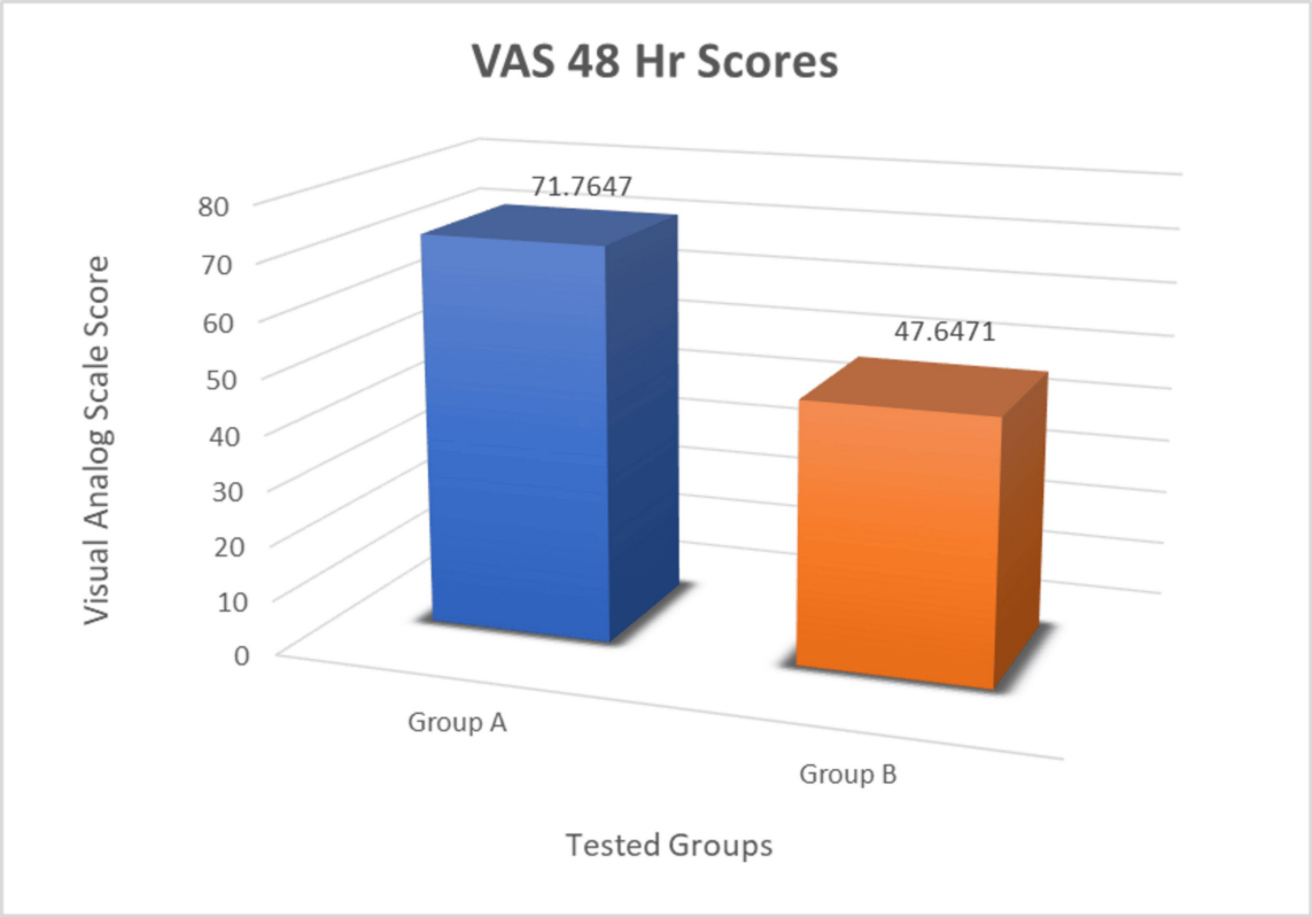
Graph of mean VAS scores of two groups VAS: visual analog scale

VAS scores were recorded at 48 hours postoperatively and the values obtained are presented in Table 2 and Figure 14. The VAS scores for the control group (Group A) after 48 hours were 71.76±5.57 and for the Experimental group (Group B) after 48 hours were 47.64±5.33. "Thus, the mean values in the experimental group were significantly lower compared to those in the control group.

**Table 2:** Comparison of mean VAS scores of two groups The statistical test applied was the Mann-Whitney U test and was highly significant at p<0.01. VAS: visual analog scale HS: highly significant

	Group	n	Mean	Std. deviation	Z value	P value
VAS 48 hour	Group A	17	71.7647	5.57370	-5.02	0.000 HS
	Group B	17	47.6471	5.33785		

Effect of ozonated water on soft tissue healing

**Figure 15:**
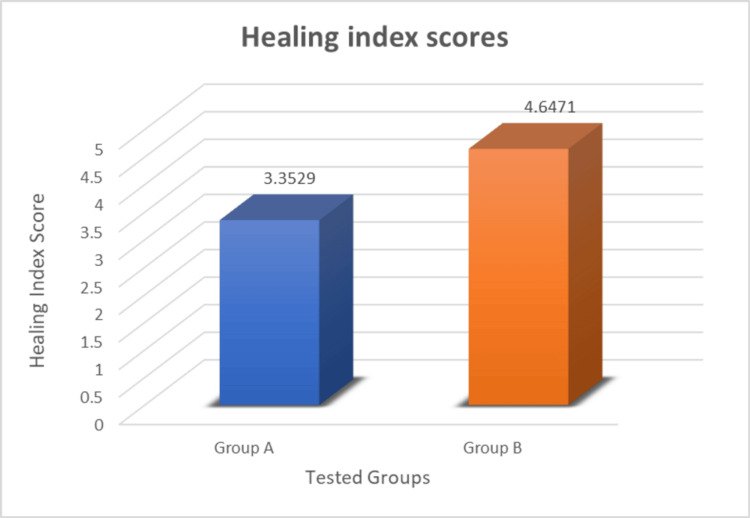
Graph of mean soft tissue healing index of two groups

Soft tissue healing was evaluated after eight days by using the tissue healing index. The mean healing index scores are presented in Table 3 and Figure 15. The mean healing index score for the control group (Group A) was 3.35±0.49 and the mean healing index score for the experimental group (Group B) was 4.64±0.49. Thus, the mean values of the tissue healing index in the experimental group were significantly higher compared to those in the control group (p<0.001).

**Table 3:** Comparison of mean soft tissue healing index of two groups The statistical test applied was the Mann-Whitney U test and was highly significant at p<0.01. HS: highly significant

	Group	n	Mean	Std. Deviation	Z value	P value
Healing index score	Group A	17	3.3529	.49259	-4.62	0.000 HS
	Group B	17	4.6471	.49259		

Effect of ozonated water on osseointegration

**Figure 16:**
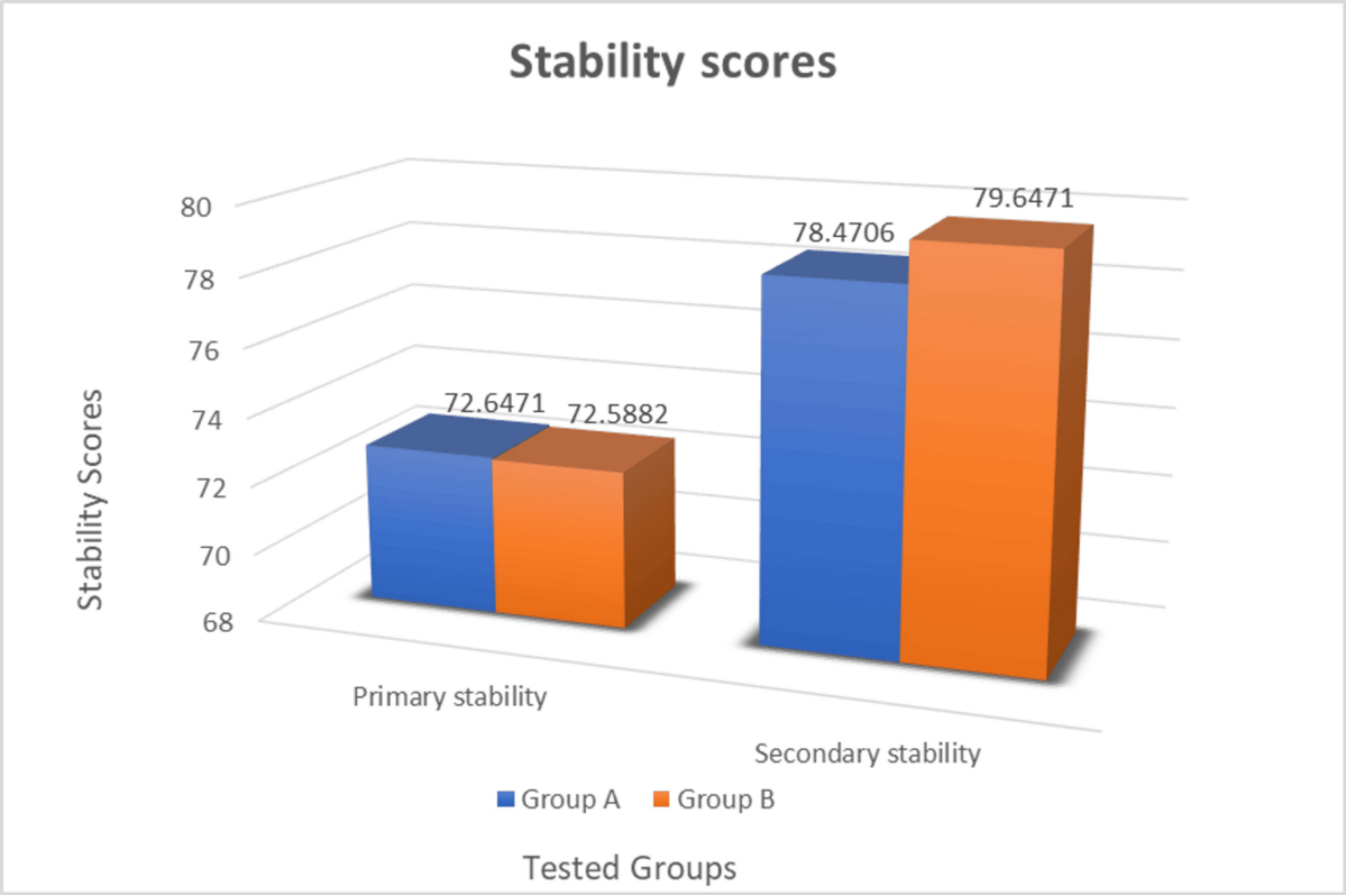
Graph of mean primary and secondary stability values of two groups

Osseointegration was assessed using RFA and the primary and secondary stability values were recorded and compared. The mean values are mentioned in Table 4 and Figure 16. The mean RFA value of the control group (Group A) for primary stability is 72.64±3.69 ISQ and secondary stability is 78.47±2.64 ISQ. The increase in stability value over the period of three months is 5.83 ISQ. The mean RFA value of the experimental group (Group B) for primary stability is 72.58±3.02 ISQ and secondary stability is 79.64±2.73 ISQ. The increase in stability value over the period of three months is 7.06 ISQ. The difference although not statistically significant, shows a slight increase in stability in the experimental group compared to that of the control group.

**Table 4:** Comparison of mean primary and secondary stability values of two groups

	Group	n	Mean	Std. deviation	Z value	P value
Primary stability	Group A	17	72.6471	3.69021	-0.01	0.98
	Group B	17	72.5882	3.02198		
Secondary stability	Group A	17	78.4706	2.64853	-1.45	0.14
	Group B	17	79.6471	2.73727		

Side effects of ozone use

No reported side effect of ozone (burning eyes, coughing, nausea, lightheadedness, and headache) was noted.

## Discussion

Ozone is a potent antioxidant agent that presents antimicrobial action and several other biological effects [9]. Even though ozone therapy has been broadly utilized in various other areas of dentistry in recent years, studies concerning the use of ozone in dental implantology have not been comprehensively compiled and evaluated. To evaluate whether ozone therapy can be helpful in the procedure of placing dental implants, the current study was undertaken.

Ozone acts as an antimicrobial (bactericidal, viricidal, and fungicidal) and possesses properties such as immunostimulating, immunomodulating, anti-inflammatory, biosynthetic, bioenergetic, anti-hypoxic, analgesic, and hemostatic to name a few [10-14].

Studies examining the benefits of ozone therapy on tissue repair have demonstrated enhancements in blood circulation (microcirculation and peripheral circulation), tissue health, and overall metabolism [15]. Ozone's hemostatic repair capabilities involve improving the blood's oxygen transport capacity, regulating antioxidant enzymes, and modulating immune cell responses, thereby enhancing resistance to oxidative stress by stimulating the antioxidant system [12].

Implants have been used to support dental restorations for many decades. However, implant placement should be carefully done to avoid the various complications that can accompany this procedure. The early postoperative period is particularly important and always depends on the soft tissue healing at the surgical site [16]. The search for therapeutic methods to alleviate systemic changes post-implant placement has also seen significant advancement [17]. Therefore, certain materials can be used to improve the rate of implant survival. Among these, ozone has been claimed to have properties that improve the clinical outcome of various surgical procedures involving the oral mucosa. Ozone therapy has received attention for its simple application, cost-effectiveness, and adaptable uses [18].

Hauser-Gerspach et al. conducted a study demonstrating that gaseous ozone effectively reduces adherent bacteria, such as *Porphyromonas gingivalis* and *Streptococcus sanguis*, from titanium and zirconia surfaces. This treatment did not affect the adhesion and proliferation of osteoblastic cells, reducing bacterial presence to below-detectable levels within 24 seconds [19].

Sunarso et al. functionalized the superhydrophilic surfaces of implants by applying ozone gas to increase osteoconductivity and reduce inflammation. After 24 hours, the contact angle of the implant surface was zero degrees. Hydroxyl groups also increased and carbon contamination decreased significantly on the implant surface. The amount of cells and cell characteristics were better in implants treated with ozone molecules [20].

The present study derived a positive correlation between a reduction in postoperative pain assessed after 48 hours and the use of ozonated water as an irrigant during the surgical procedure of implant placement. This was found perhaps due to the suppressing effect of ozone on the production of mediators of inflammation and subsequent decrease in pain. These results are in conjunction with the results of research conducted by Karaca et al. where mean pain VAS scores for the ozonated group of patients were observed to be significantly lower compared to other groups at day one and day three of follow-up [6].

The second objective was to evaluate the soft tissue healing around the dental implants, eight days after the surgical procedure. The results observed suggest that the use of ozonated water as an irrigant significantly improves the soft tissue healing of the surgical site. The effect of ozone is to promote hemostasis and enhance the release of growth factors. Moreover, it inhibits microbial proliferation and regulates antioxidant enzymes. These results are in agreement with a study performed by Sghaireen et al. to assess the benefit of topical ozone therapy on wound healing and pain after dental implant placement. The study results showed that topical ozone treatment improved soft tissue healing one and two days after implant placement [21].

Another objective was to compare osseointegration between the control and experimental groups by evaluating primary and secondary stability using the ISQ value. The results did not show significant improvement in the secondary stability values of the experimental group. However, a slight increase in the difference between the primary and secondary stability of the experimental group was noted compared to the control group. Ozone therapy enhances the peri-implant interface environment through its bactericidal properties, boosting local oxygen supply and facilitating hemostasis [22]. This, in turn, is believed to stimulate osteoblast proliferation, thereby accelerating bone formation and mineralization at the peri-implant bone interface. A study conducted by Hadary et al. shows a significant difference in osseointegration of implants placed in the tibia of rabbits who received topical ozonated oil treatment compared to those who did not. In ozonated specimens collected from the ozonated oil-treated group (Group A), intimate contact between the surface of the implant and the newly formed mature bone was observed [23]. The variability in findings concerning osseointegration arises due to the multifaceted nature of bone healing, where outcomes manifest over an extended period. Furthermore, the assessment of osseointegration lacks complete objectivity, as the most accurate method is a histomorphometric examination, necessitating bone resection at the implant site, which is practically unfeasible in human subjects [24].

The limitations of the study include the restricted number of follow-up periods, due to which minimal changes happening concurrently could not be recognized. The ozonated water was used at only one fixed concentration so the effect of varied concentrations on the clinical outcomes could not be assessed. Other outcomes like marginal bone loss, changes in bone density could not be studied. Radiological examination would have aided in determining these outcomes. The systemic anti-inflammatory effect of using ozone could have been evaluated by conducting preoperative and postoperative blood investigations such as C-reactive protein (CRP), erythrocyte sedimentation rate (ESR), WBC count, and serum amyloid A (SAA). To achieve sufficient statistical power, cross-arch studies may require larger sample sizes, especially if there is significant variability between the arches being compared. If different arches are assessed by different observers or under different conditions, there is a potential for detection bias, where the outcomes might be influenced by the differences in observation rather than the intervention itself.

Further studies are imperative to investigate varied concentrations and forms of ozone, alongside employing advanced technologies, which can elucidate its broader impact on bone healing and osseointegration. Moreover, integrating diverse methodologies will contribute to a better understanding of its therapeutic potential and pave the way for optimized clinical applications.

## Conclusions

Within the limitations of the study, we came to the conclusion that there was a significant decrease in postoperative pain in implants placed using ozonated water as the irrigant during implant site osteotomy as evaluated 48 hours after the procedure. There was significantly enhanced soft tissue healing surrounding the dental implants placed using ozonated water as the irrigant as evaluated eight days after the surgical procedure. When the difference in the primary and secondary stability was calculated, a significant increase in the stability values of the ozonated group was not found.

Hence, the utilization of ozonated water as an irrigant shows the potential to enhance short-term outcomes related to postoperative pain and soft tissue healing following implant placement. However, its impact on long-term aspects such as osseointegration and bone healing appears to be inconsequential. While further research may be warranted to explore potential applications in other aspects of implant dentistry, the current findings suggest that ozonated water's benefits are primarily confined to the soft tissue realm in the context of implant procedures.

## References

[1] Guillaume B (2016). Dental implants: a review. Morphologie.

[2] Nolan R, Kemmoona M, Polyzois I, Claffey N (2014). The influence of prophylactic antibiotic administration on post-operative morbidity in dental implant surgery. A prospective double blind randomized controlled clinical trial. Clin Oral Implants Res.

[3] Kohl BA, Deutschman CS (2006). The inflammatory response to surgery and trauma. Curr Opin Crit Care.

[4] Jorgen BD, Kehlet H (2006). Postoperative pain and its management. Wall and Melzack’s Textbook of Pain.

[5] Albrektsson T, Brånemark PI, Hansson HA, Lindström J (1981). Osseointegrated titanium implants. Requirements for ensuring a long-lasting, direct bone-to-implant anchorage in man. Acta Orthop Scand.

[6] Karaca IR, Ergun G, Ozturk DN (2018). Is Low-level laser therapy and gaseous ozone application effective on osseointegration of immediately loaded implants?. Niger J Clin Pract.

[7] Bocci VA (2006). Scientific and medical aspects of ozone therapy. State of the art. Arch Med Res.

[8] Rakovsky S, Zaikov G (2009). Application of ozone in medicine. Chem Technol.

[9] Seidler V, Linetskiy I, Hubálková H (2008). Ozone and its usage in general medicine and dentistry. A review article. Prague Med Rep.

[10] Broadwater WT, Hoehn RC, King PH (1973). Sensitivity of three selected bacterial species to ozone. Appl Microbiol.

[11] Elvis AM, Ekta JS (2011). Ozone therapy: a clinical review. J Nat Sci Biol Med.

[12] Grootveld M, Chang H, Grootveld M (2012). Oxidative consumption of oral biomolecules by therapeutically-relevant doses of ozone. Adv Chem Eng Sci.

[13] Sagai M, Bocci V (2011). Mechanisms of action involved in ozone therapy: is healing induced via a mild oxidative stress?. Med Gas Res.

[14] Margalit M, Attias E, Attias D, Elstein D, Zimran A, Matzner Y (2001). Effect of ozone on neutrophil function in vitro. Clin Lab Haematol.

[15] Stübinger S, Sader R, Filippi A (2006). The use of ozone in dentistry and maxillofacial surgery: a review. Quintessence intl.

[16] Myshin HL, Wiens JP (2005). Factors affecting soft tissue around dental implants: a review of the literature. J Prosthet Dent.

[17] Khouly I, Braun RS, Ordway M, Alrajhi M, Fatima S, Kiran B, Veitz-Keenan A (2021). Post-operative pain management in dental implant surgery: a systematic review and meta-analysis of randomized clinical trials. Clin Oral Investig.

[18] Matar IM, El-Sharkawy AM, Mohamed NS, Mawsouf MN (2016). Clinical and radiographic evaluation the effect of ozone therapy on tissue surrounding implant retained mandibular overdentures. Ozone Therapy Glob J.

[19] Hauser-Gerspach I, Vadaszan J, Deronjic I (2012). Influence of gaseous ozone in peri-implantitis: bactericidal efficacy and cellular response. An in vitro study using titanium and zirconia. Clin Oral Investig.

[20] Sunarso Sunarso, Toita R, Tsuru K, Ishikawa K (2016). A superhydrophilic titanium implant functionalized by ozone gas modulates bone marrow cell and macrophage responses. J Mater Sci Mater Med.

[21] Sghaireen MG, Alzarea BK, Alduraywish AA (2020). Effect of aqueous ozone solution irrigation on healing after treatment with dental implants: a cross-over randomized controlled clinical trial. J Hard Tissue Biol.

[22] Kashperskii YP, Adamyan AA, Makarov VA (1995). Study of the hemostatic properties of gaseous ozone. Bull Exp Biol Med.

[23] El Hadary AA, Yassin HH, Mekhemer ST, Holmes JC, Grootveld M (2011). Evaluation of the effect of ozonated plant oils on the quality of osseointegration of dental implants under the influence of Cyclosporin A an in vivo study. J Oral Implantol.

[24] Park YS, Yi KY, Lee IS, Jung YC (2005). Correlation between microtomography and histomorphometry for assessment of implant osseointegration. Clin Oral Implants Res.

